# Assessment of Single Cell RNA-Seq Normalization Methods

**DOI:** 10.1534/g3.117.040683

**Published:** 2017-05-03

**Authors:** Bo Ding, Lina Zheng, Wei Wang

**Affiliations:** * Department of Chemistry and Biochemistry, University of California at San Diego, La Jolla, California 92093-0359; † Department of Cellular and Molecular Medicine, University of California at San Diego, La Jolla, California 92093-0359

**Keywords:** normalization, scRNA, statistical index

## Abstract

We have assessed the performance of seven normalization methods for single cell RNA-seq using data generated from dilution of RNA samples. Our analyses showed that methods considering spike-in External RNA Control Consortium (ERCC) RNA molecules significantly outperformed those not considering ERCCs. This work provides a guidance of selecting normalization methods to remove technical noise in single cell RNA-seq data.

Single cell RNA-seq (scRNA-seq) is becoming a powerful tool to study many biological problems, such as tissue heterogeneity (Kumar *et al.* 2014; Patel *et al.* 2014; Usoskin *et al.* 2015), and cell differentiation during development (Biase *et al.* 2014; Trapnell *et al.* 2014; Treutlein *et al.* 2014). Normalization is particularly critical for interpreting scRNA-seq data, including detection of differentially expressed gene, and identification of cell subtypes (Stegle *et al.* 2015). The small amount of samples used in scRNA-seq often leads to higher technical noise compared to bulk RNA-seq, which needs to be removed. Various methods have been developed for normalization of scRNA-seq data, including fragments per kilobase of transcript per million mapped reads (FPKM) (Mortazavi *et al.* 2008), upper quartile (UQ) (Bullard *et al.* 2010), trimmed mean of *M*-values (TMM) (Robinson and Oshlack 2010), DESeq (Love *et al.* 2014), removed unwanted variation (RUV) (Risso *et al.* 2014), and gamma regression model (GRM) (Ding *et al.* 2015). Most of these methods were developed for bulk RNA-seq, and then applied directly to scRNA-seq analysis. The performance of these methods was assessed on bulk (Dillies *et al.* 2013; Risso *et al.* 2014), but not on scRNA-seq, data because the ground truth is likely unknown for single cell assays.

Recently, the NIH Single Cell Analysis Program—Transcriptome Project (SCAP-T) collected two well-characterized human reference RNA samples: Universal Human Reference RNA (UHR) and Human Brain Reference (HBR) (Dueck *et al.* 2016). A set of RNA-seq data was generated using different amount of RNAs obtained from dilution (10 ng considered as bulk, 100 and 10 pg). These samples were prepared for sequencing using three protocols: antisense RNA IVT protocol (abbreviated as aRNA or A) (Morris *et al.* 2011), a customized C1 SMARTer protocol performed on a Fluidigm C1 94-well chip (S) (Ramskold *et al.* 2012), and a modified NuGen Ovation RNA sequencing protocol (N) (Kurn *et al.* 2005) (Table 1). These data can be divided into six groups based on the sample source and amount: UHR, bulk; UHR, 100 pg; UHR, 10 pg; HBR, bulk; HBR, 100 pg, and HBR 10 pg. It is natural to assume that 100 pg samples would be more similar to bulk than 10 pg samples. Therefore, this set of data is invaluable to compare normalization methods because the ground truth is known. Furthermore, 51 of these samples were mixed with spike-in ERCC RNA molecules, which makes it possible to evaluate the impact of considering ERCCs in normalization of scRNA-seq data.

**Table 1:** Dilution RNA-seq data generated by SCAP-T

Single Cell Protocol	Human Brain Reference RNA Amount (HBR)			Universal Human Reference RNA Amount (UHR)			Total
	10 pg	100 pg	Bulk	10 pg	100 pg	Bulk	
aRNA (A)	18(5)*^a^*	3	—	12	7	—	40
C1 SMARTer (S)	15(15)	5(5)	—	15(15)	5(5)	—	40
Nugen Ovation RNASeq V2 (N)	4	4	—	15	11	—	34
None	—	—	3	—	—	3	6
Total	37	12	3	42	23	3	120

a The number in the parenthesis is the number of samples containing spike-in ERCCs.

Using this data set, we have assessed the performance of several commonly used normalization methods: FPKM, upper quartile (UQ), trimmed mean of *M*-values (TMM), DESeq, removed unwanted variation (RUV), and gamma regression model (GRM). This study thus provides a guidance of choosing normalization methods to remove technical noise in single cell RNA-seq data.

## Materials and Methods

### Selection of variable genes

Before applying different normalization methods on the data, we first selected variable genes using the following criteria: (1) across the 114 nonbulk samples, at least two samples with log(fpkm) >2; (2) across 114 nonbulk samples, variant of log(fpkm) >1. In this way, we found 13,375 genes for the following analyses.

### Running normalization methods

FPKM (Garber *et al.* 2011) normalizes the gene counts in consideration of library size and gene length. The UQ (Bullard *et al.* 2010) and TMM (Robinson and Oshlack 2010) methods are implemented in the edgeR Bioconductor package. The DESeq (Anders and Huber 2010) method is implemented in the Bioconductor package. For all methods, we ran the R function using default parameters. Remove unwanted variants (RUV) (Risso *et al.* 2014) is included in the RUVnormalize Bioconductor packages. The model sets up a generalized linear regression model between observed RNA-seq read counts and known covariates of interest, along with unknown unwanted variation factors. Different options of RUV, RUVr, and RUVg have different ways to estimate the unwanted variant factors. RUVr uses residuals from a first-pass regression of read counts (Risso *et al.* 2014), and considers the least differentially expressed genes across samples. RUVg considers negative controls such as External RNA Control Consortium (ERCC) spike-ins, and assumes that they are not differentially expressed across samples. All the R functions were run using default parameters. For empirical undifferentially expressed genes in running RUVr, we chose genes not in the top 6000 differentially expressed genes. GRM (Ding *et al.* 2015) fits a gamma regression model between FPKM values of reads and the concentration of ERCC spike-ins, and then make estimates of the molecular concentration of the genes from the reads.

### Assessment of clustering performance

After normalization, we clustered the normalized gene reads using hierarchical clustering with the Ward method, and the metric was Pearson correlation between normalized gene reads. We assessed the clustering results using the following statistical indices:

#### Rand index:

The Rand index (Rand 1971) evaluates the correctness of clustering using prior labels. Given a set of *n* elements *S* = $\left\{s_{1},s_{2},\cdots ,s_{n}\right\},$ and two partitions needed to be compared, a partition of *S* into *M* clusters $X=\left\{X_{1},X_{2},\cdots ,X_{M}\right\},$ and a partition of *S* into *N* clusters $Y=\left\{Y_{1},Y_{2},\cdots ,Y_{N}\right\},$ for certain $1\leq i,j\leq n\left(i\neq j\right),1\leq k,k_{1},k_{2}\leq M\left(k_{1}\neq \;k_{2}\right),1\leq l,l_{1},l_{2}\leq N\left(l_{1}\neq \;l_{2}\right),$ a,b,c,and d are defined as follows:

$$\{\begin{array}{c} a=\left|S^{a}\right|,\mathrm{where}\;S^{a}=\left\{\left(s_{i},s_{j}\right)|s_{i},s_{j}\in X_{k},s_{i},s_{j}\in Y_{l}\right\}\\ b=\left|S^{b}\right|,\mathrm{where}\;S^{b}=\left\{\left(s_{i},s_{j}\right)|s_{i}\in X_{k_{1}},s_{j}\in X_{k_{2}},s_{i}\in Y_{l_{1}},s_{j}\in Y_{l_{2}}\right\}\\ c=\left|S^{c}\right|,\mathrm{where}\;S^{c}=\left\{\left(s_{i},s_{j}\right)|s_{i},s_{j}\in X_{k},s_{i}\in Y_{l_{1}},s_{j}\in Y_{l_{2}}\right\}\\ d=\left|S^{d}\right|,\mathrm{where}\;S^{d}=\left\{\left(s_{i},s_{j}\right)|s_{i}\in X_{k_{1}},s_{j}\in X_{k_{2}},s_{i},s_{j}\in Y_{l}\;\right\} \end{array}$$

Then Rand index can be computed as follows:

$$R=\frac{a+b}{a+b+c+d}$$

where (*a* + *b*) is the number of agreements between *X* and *Y*, while (*c* + *d*) is the number of disagreements between *X* and *Y*. The higher the Rand index, the more similar the two partitions. As we compared the clustering partitions to the prior known labels, the higher Rand index indicates the better the clustering is.

#### Dunn index:

The Dunn index (Dunn 1974) evaluates the compactness and separation of the clustering. Specifically, given a set of *n* elements *S* = $\left\{s_{1},s_{2},\cdots ,s_{n}\right\},$ and the clustering partition as $\zeta =\left\{C_{1},C_{2},\cdots ,C_{K}\right\},$ the Dunn index is computed as follows:

$$D\left(\zeta \right)=\frac{\begin{array}{c} \min\\ C_{p},C_{q}\in \zeta ,C_{p}\neq C_{q} \end{array}\left(\begin{array}{c} \min\\ i\in C_{p},\;j\in C_{q} \end{array}\mathrm{dist}\left(i,j\right)\right)}{\begin{array}{c} \max\\ C_{m}\in \zeta \end{array}\mathrm{diam}\left(C_{m}\right)}$$

By definition, the higher the Dunn index, the better the clustering, considering enough separation compared to diameter of individual clusters. Here, the distance between two samples is defined as 1 − the Pearson correlation coefficient between two samples.

#### Jaccard index:

The Jaccard index evaluates the stability of clustering that measures the similarity between two finite subsets (Jaccard 1912a; Tan *et al.* 2006). Given two subsets, *A* and *B*, the Jaccard index is computed as:

$$J\left(A,B\right)=\frac{\left|A\cap^{\text{​}}B\right|}{\left|A\cup^{\text{​}}B\right|}$$

We used the Flexible Procedures for Clustering (fpc) package in R to calculate the Jaccard index, with random subsetting without replacement of samples. Each time a subset of samples was drawn, we clustered the samples, and then computed the maximum Jaccard index between the new cluster and the prior known cluster. Repeating *B* times for drawing *B* random subsets (here we chose *B* = 100), we then computed the mean of all the maximum Jaccard index (Hennig 2007). The higher the Jaccard index, the more robust the clustering.

### Data availability

The authors state that all data necessary for confirming the conclusions presented in the article are represented fully within the article.

## Results and Discussion

We selected seven methods to normalize the data of the 120 RNA-seq samples. These methods fall into two categories, depending on whether or not spike-in ERCC molecules were taken into consideration in normalization.

### Evaluation of the methods not considering ERCC

**Figure 1:**
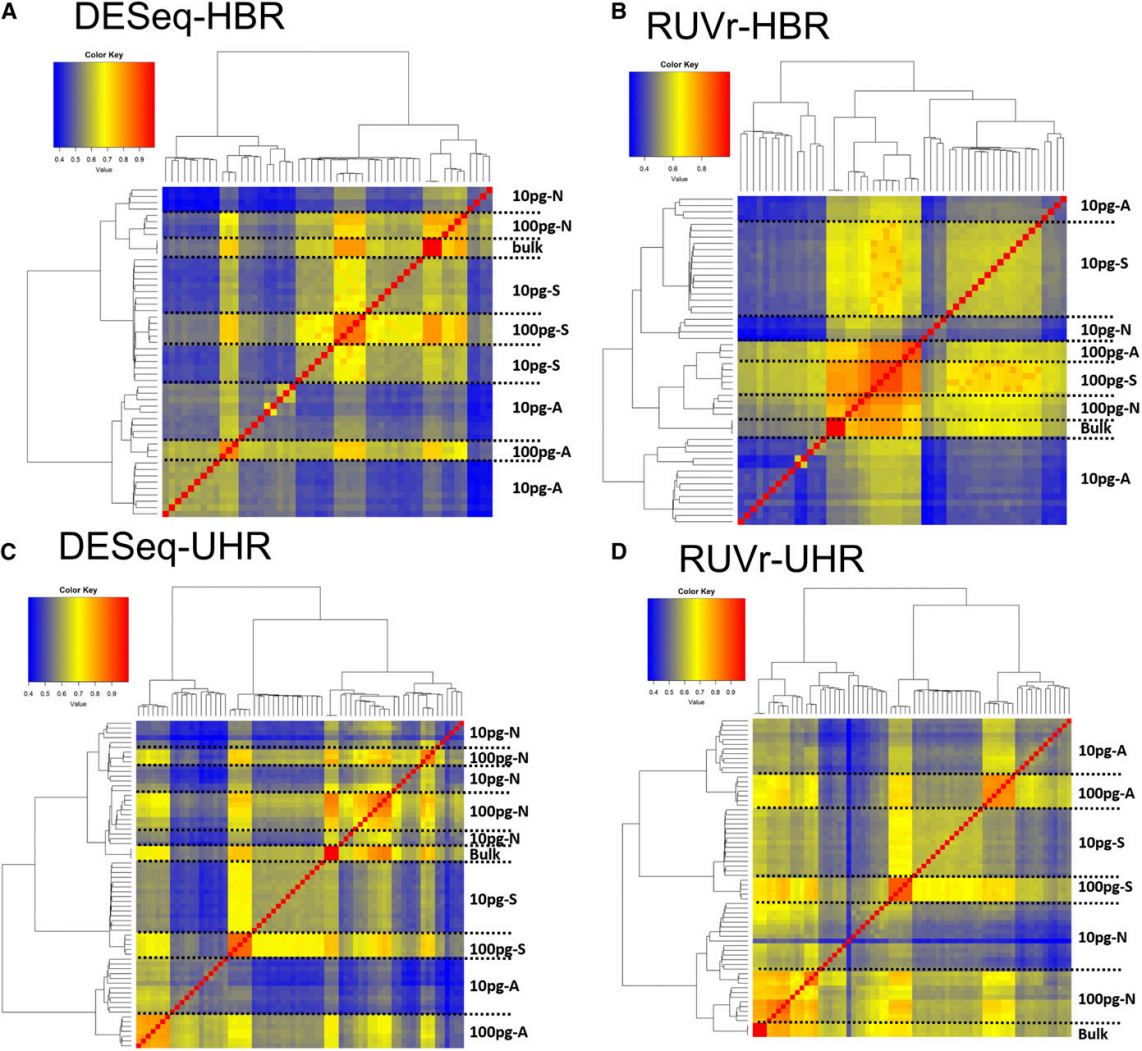
Hierarchical clustering of HBR and UHR RNA-seq samples with different normalization methods not considering ERCC. (A) DESeq with HBR samples; (B) RUVr with HBR samples; (C, D) DESeq and RUVr with UHR samples. The results of other methods are shown in Figure S1 in File S1. A, aRNA; N, Nugen Ovation RNASeq V2; S, C1 SMARTer.

We first evaluated the performance of five methods not considering ERCCs: FPKM (Mortazavi *et al.* 2008), UQ (Bullard *et al.* 2010), TMM (Robinson and Oshlack 2010), DESeq (Love *et al.* 2014), and RUVr (Risso *et al.* 2014) (nonconsidering ERCC version of RUV) (see *Materials and Methods* for the details of setup for running each method). A total of 13,375 genes was selected with log(fpkm) >2 in at least two of the 114 nonbulk samples (100 or 10 pg). Using Pearson correlation as the similarity metric, we clustered all 120 samples (114 nonbulk and six bulk samples) using hierarchical clustering (Figure 1 and Supplemental Material, Figure S1 in File S1). For all methods, the UHR and HBR samples were well separated as expected, and we thus focused on evaluating how well the HBR and UHR samples were further clustered. In either the UHR or HBR group, samples normalized by FPKM, UQ, TMM, and DESeq tended to cluster by sequencing protocol rather than RNA amount, which indicates that the differences introduced by the protocols were not completely removed by normalization. In contrast, the HBR samples normalized by RUVr were largely clustered by RNA amount rather than sequencing protocol, although a significant portion of 10 pg samples sequenced using aRNA were clustered separately (Figure 1 and Figure S1 in File S1). Qualitatively, RUVr normalization alleviates the difference between scRNA-seq protocols and the clustering results are closer to the ground truth than the other methods, *i.e.*, the samples are clustered based on the source (HBR) and the RNA amount (bulk, 100 or 10 pg). However, the UHR samples normalized with RUVr were still clustered according to protocols rather than RNA amount. The other four methods showed worse clustering results than RUVr because 10 and 100 pg samples were mixed in each protocol group (Figure 1 and Figure S1 in File S1).

**Figure 2:**
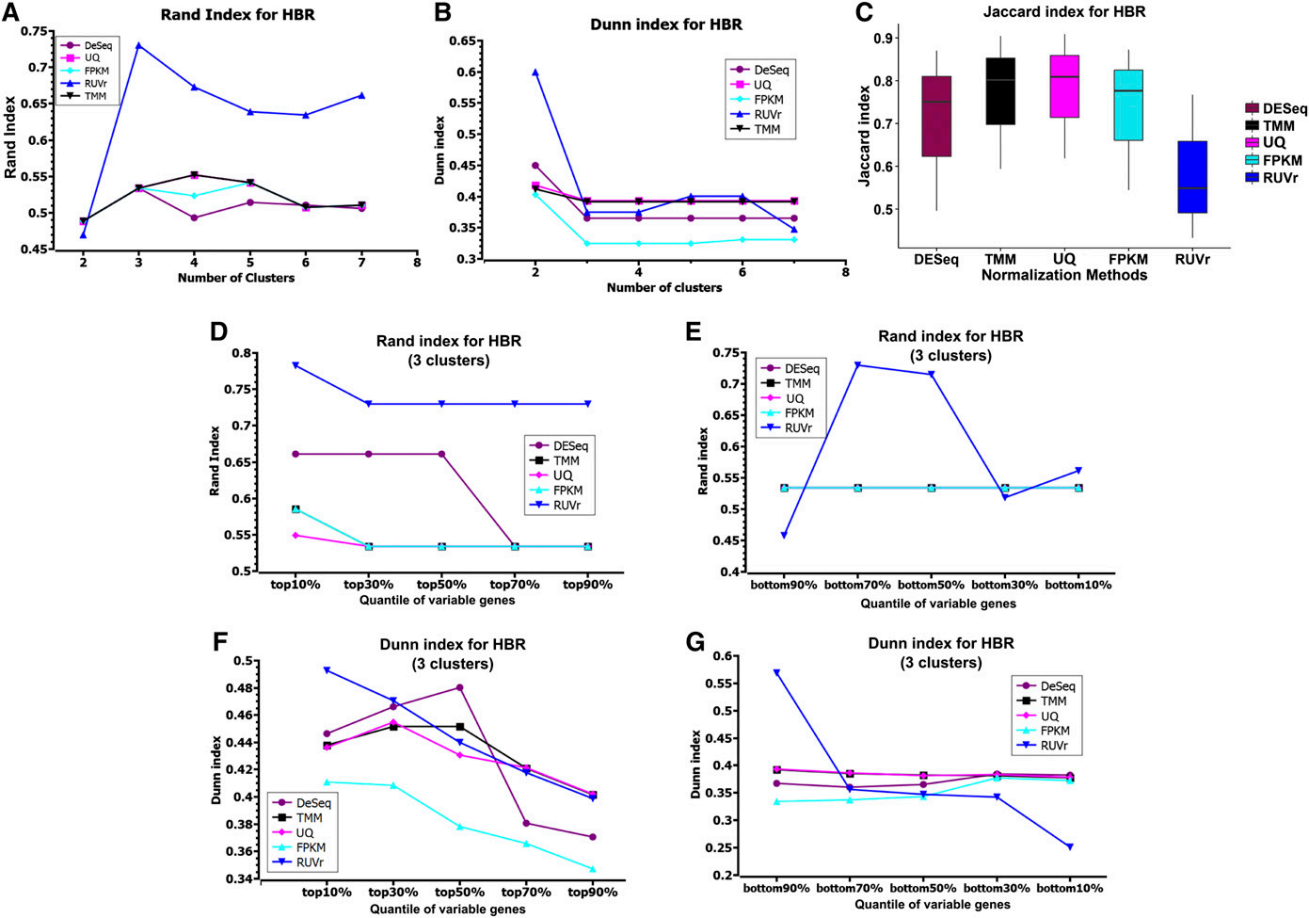
Comparison of five normalization methods not using ERCC on the HBR samples. (A) Rand index with different number of clusters; (B) Dunn index with different number of clusters; (C) Jaccard index with three clusters; (D) Rand index with the most variable genes; (E) Rand index with the least variable genes; (F) Dunn index with the most variable genes; and (G) Dunn index with the least variable genes. (D–G) Results using three clusters as the ground truth (bulk, 100 and 10 pg HBR samples). UHR results are shown in the supplementary materials (Figure S2 in File S1).

To quantify the similarity between the clusters generated from different normalization methods and the ground truth, we cut the clusters at different hierarchy levels, and calculated the Rand index between the clusters and the ground truth, which reflects the correctness of clustering. The Rand index is the ratio between the sample pairs that are correctly clustered and the total sample pairs (Rand 1971). Because all the methods successfully separated HBR and UHR samples, as discussed above, we calculated the Rand index on HBR and UHR samples separately. For either the HBR or UHR samples, we varied the cutoffs to cut the hierarchical trees into two to seven clusters, and calculated Rand index for each cutoff (Figure 2A and Figure S2 in File S1). RUVr clearly outperformed the other methods.

Next, we evaluated the compactness and separation of clusters using the Dunn index, which is the ratio of the smallest distance between clusters to the largest intracluster distance, and the distance between the two samples is defined as 1 − the Pearson correlation between the samples (Dunn 1973). We computed the Dunn index on HBR and UHR samples separately. RUVr had a much higher Dunn index than the other methods when all the samples were clustered into two groups. For more clusters, all the methods had comparable Dunn index. This observation suggests that the separation between clusters is insensitive to normalization methods when the 120 samples were clustered into more than three clusters.

It is important to examine whether the clustering results are robust to the selection of samples and genes. We first divided HBR or UHR samples into three clusters: bulk, 100 and 10 pg. Then, we randomly selected 50% of the total samples, clustered them into three groups using the 13,375 genes selected above. We computed the maximum Jaccard index (Jaccard 1912b) (see definition in *Materials and Methods*) between the new cluster and the ground truth. The Jaccard index reflects the correctly clustered samples using the subsets of samples, and thus the robustness of the clustering. All methods except RUVr showed similar Jaccard index values. Next, to evaluate the robustness of clustering to genes used to calculate Pearson correlation coefficient between samples, we ranked the 13,375 genes using their coefficients of variance (CV) (Brennecke *et al.* 2013), which is the standard deviation of gene expression divided by the mean, reflecting the variation of a gene’s expression across samples. We selected 10 sets of genes: top 10%, top 30%, top 50%, top 70%, top 90%, and bottom 10%, bottom 30%, bottom 50%, bottom 70%, and bottom 90%. In general, using the most variable genes achieved the most correct clustering, *i.e.*, high Rand index, and using a sufficient number of the most variable genes (top 10% for RUVr and FPKM, top 30% for UQ and TMM, and top 50% for DeSeq) gave the highest Dunn index, which represents the best separation of the clusters (Figure 2, D–G). Using the least variable genes (most invariable genes), such as the bottom 10% variable genes, normally showed lower Rand and Dunn index than using more variable genes, such as the bottom 90% variable genes (Figure 2, D–G).

### Evaluation of methods considering ERCC

**Figure 3:**
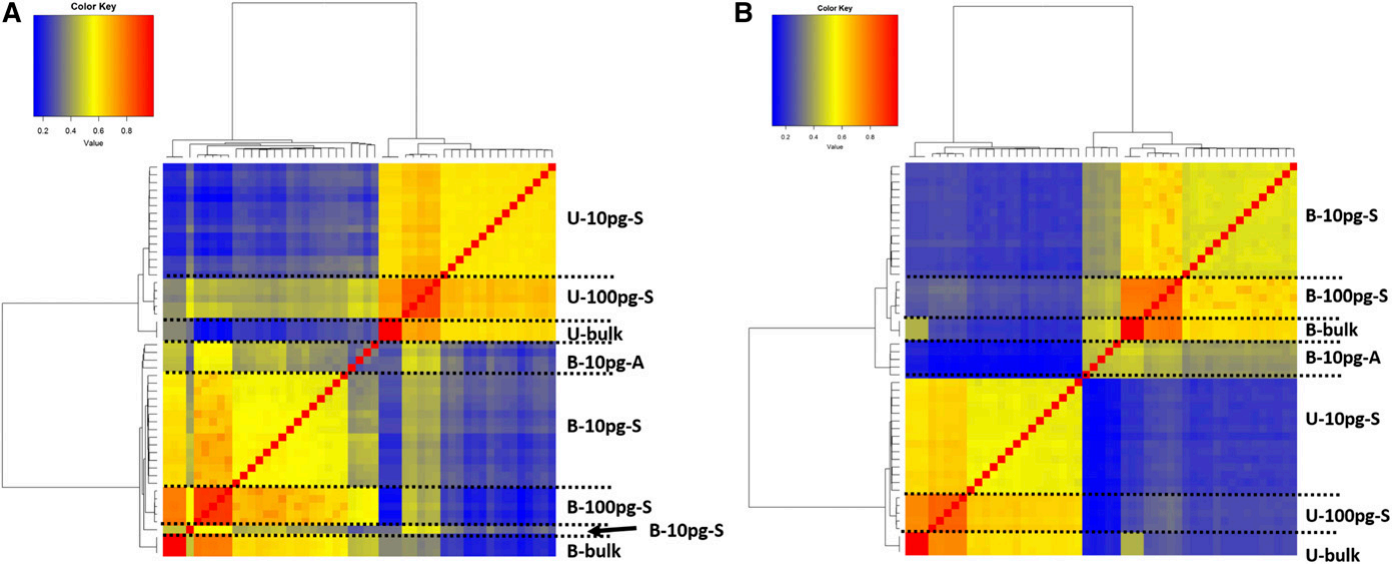
Hierarchical clustering of 51 samples with different normalization methods using ERCC. (A) RUVg; (B) GRM. B, HBR; U, UHR; A, aRNA; S, C1 SMARTer.

We next evaluated the performance of two methods considering ERCCs: RUVg (RUV model considering ERCC) (Risso *et al.* 2014) and GRM (Ding *et al.* 2015) (see *Materials and Methods* for the details of the setup for running each method). Among the 120 RNA-seq runs, 45 samples containing spike-in ERCCs were normalized using the two methods, and then clustered with six bulk samples together (Figure 3). The clustering results were similar to those obtained using the 13,375 selected genes. UHR and HBR samples were clearly separated, and the samples with the same amount of RNA were largely clustered together. One outlier in the RUVg cluster is a HBR 10 pg sample sequenced using the aRNA protocol that was clustered with HBR 100 pg samples.

**Figure 4:**
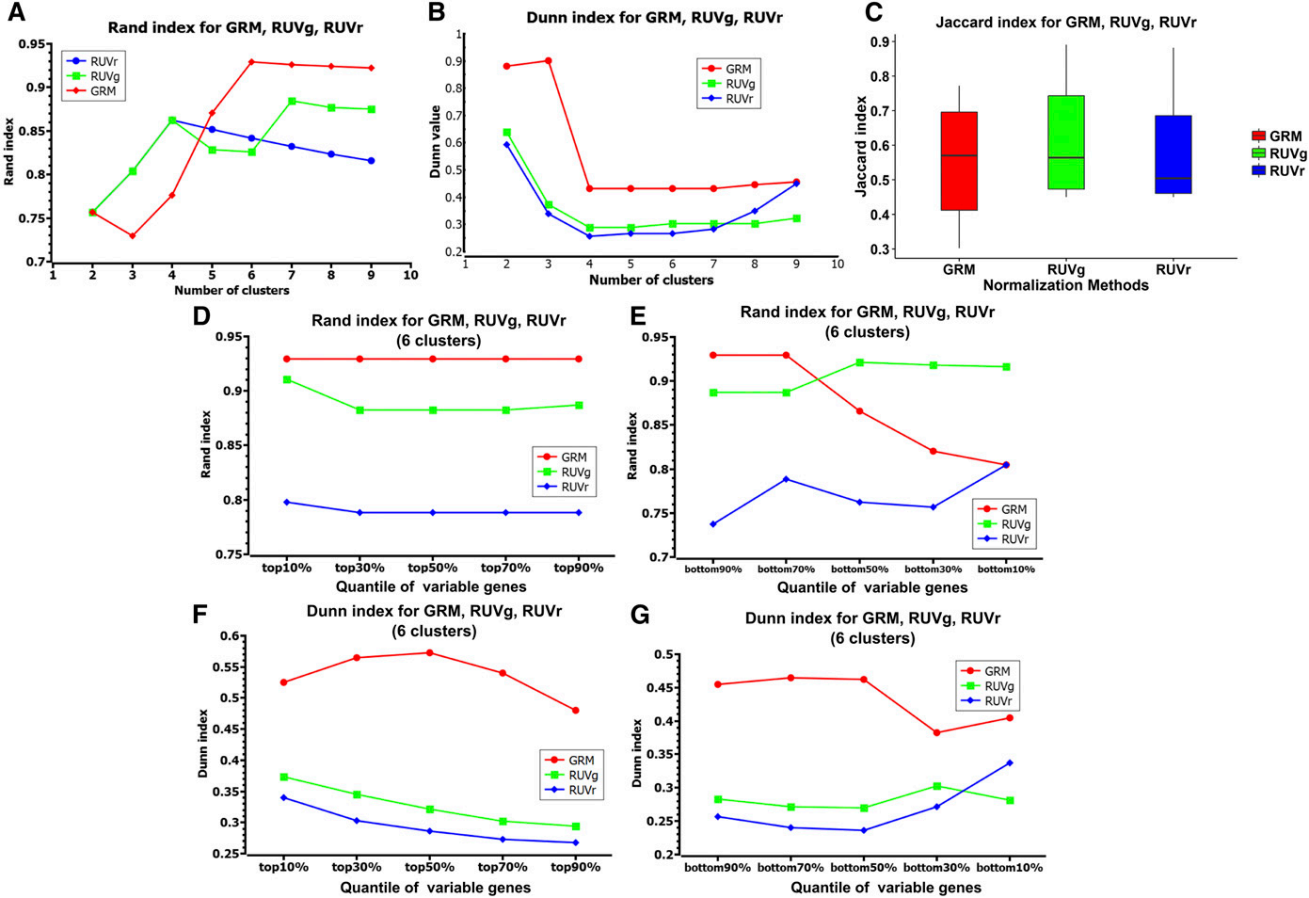
Comparison of two normalization methods using ERCC (RUVg and GRM) and one not using ERCC (RUVr). (A) Rand index with different number of clusters; (B) Dunn index with different number of clusters; (C) Jaccard index with six clusters; (D) Rand index with the most variable genes; (E) Rand index with the least variable genes; (F) Dunn index with the most variable genes; and (G) Dunn index with the least variable genes. (D–G) Results of using six clusters as the ground truth (bulk, 100 and 10 pg of HBR and UHR samples).

**Figure 5:**
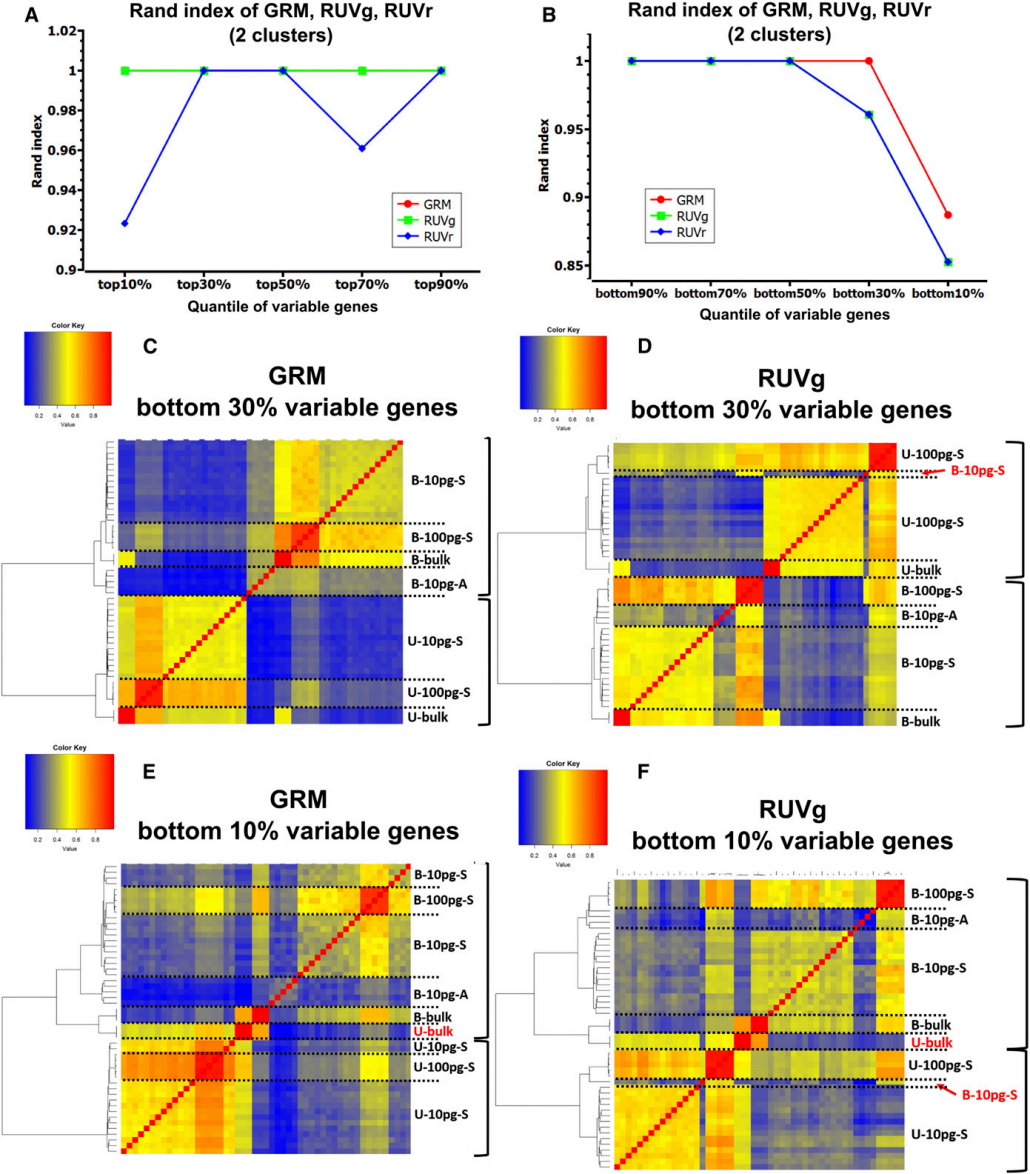
Comparison of two normalization methods using ERCC (RUVg and GRM) and one not using ERCC (RUVr) if the ground truth has two clusters (HBR and UHR samples). (A) Rand index of GRM, RUVg and RUVr with the most variable genes. (B) Rand index of GRM, RUVg and RUVr with the least variable genes. (C, D) Hierarchical clustering of GRM and RUVg using the bottom 30% variable genes. (E, F) Hierarchical clustering of GRM and RUVg using the bottom 10% variable (the most invariable) genes. Right brackets label the first branches. Red samples are wrongly clustered samples.

The GRM showed a slightly higher Rand index than RUVg when the number of clusters was the same as the ground truth (6) or more (Figure 4A). GRM also had a better Dunn index and comparable Jaccard index (Figure 4, B and C), which suggests that GRM achieves better separation of clusters (Dunn index). The robustness to sample selection is comparable for the two methods, as indicated by their similar Jaccard index. Furthermore, we assessed how sensitive the two methods are to gene selection. For the Dunn index, GRM outperformed RUVg, regardless of whether the most variable, or the least variable, genes were selected (Figure 4, F and G). For the Rand index, using the most variable genes, GRM consistently showed higher values than RUVg; using the least variable genes, GRM achieved higher Rand index using the bottom 90 and 70% genes, but lower values using the bottom 50, 30 or 10% genes than RUVg and RUVr (six clusters of bulk, 100 and 10 pg samples for HBR and UHR were considered as the ground truth) (Figure 4, D and E). We examined the clusters generated using the bottom 30% variable genes. GRM still correctly clustered all the samples, but RUVg incorrectly clustered some HBR samples with UHR, despite a higher Rand index (Figure 4E and Figure 5). We then used the bottom 10% variable genes (the most invariable genes) for a further comparison. Both methods misclustered UHR bulk with the HBR samples, but RUVg had additional HBR samples mistakenly clustered with UHR samples (Figure 5). For the Jaccard index, GRM performed better than RUVg, and RUVr with genes selected from top 10% through top 90% and bottom 90%, bottom 70% and bottom 50%. When the least variable genes were selected (bottom 10%), RUVg and RUVr significantly outperformed GRM (Figure S3 in File S1).

Notably, both methods considering ERCC outperformed the methods not considering ERCC on the 51 samples (45 10 or 100 pg and six bulk samples) containing spike-in ERCCs (Figure 4). RUVr, as a representative method, largely outperforms the other methods not considering ERCCs. Obviously, both GRM and RUVg showed higher Rand, Dunn and Jaccard index than RUVr, which indicates the value of considering ERCC in normalization and removing noise from scRNA-seq. Normalization of bulk RNA-seq using ERCCs can be useful, but may be less critical because the technical noise is smaller in bulk RNA-seq than in scRNA-seq.

### Conclusions

Here, we evaluated the performance of seven normalization methods on 120 RNA-seq runs in terms of correctness (Rand index), compactness (Dunn index), and robustness (Jaccard index, robustness analysis using different sets of samples and genes) of clustering. The results showed that, for methods not considering spike-in ERCCs, RUVr showed higher Rand index and lower Jaccard index than FPKM, UQ, DESeq, and TMM; all methods showed similar Dunn index values. Considering ERCC, such as in the models of RUVg and GRM, significantly improved performance. Between RUVg and GRM, GRM is more robust in terms of selecting different sets of genes that generate similar clusters. Spike-in ERCCs would reduce the sequencing depth of mRNAs of interest, and there is also a concern about whether the synthesized ERCC molecules behave the same as mRNAs from the cell. Our analyses suggest that calibration of scRNA-seq to the spike-in ERCC is a powerful means of removing technical noise when the ERCCs are correctly modeled.

## Supplementary Material

Supplemental material is available online at www.g3journal.org/lookup/suppl/doi:10.1534/g3.117.040683/-/DC1.

## References

[bib1] Anders S. Huber W., 2010 Differential expression analysis for sequence count data. Genome Biol. 11: R106. 20979621 10.1186/gb-2010-11-10-r106 PMC3218662

[bib2] Biase F. H. Cao X. Zhong S., 2014 Cell fate inclination within 2-cell and 4-cell mouse embryos revealed by single-cell RNA sequencing. Genome Res. 24: 1787–1796. 25096407 10.1101/gr.177725.114 PMC4216920

[bib3] Brennecke P. Anders S. Kim J. K. Kolodziejczyk A. A. Zhang X., 2013 Accounting for technical noise in single-cell RNA-seq experiments. Nat. Methods 10: 1093–1095. 24056876 10.1038/nmeth.2645

[bib4] Bullard J. H. Purdom E. Hansen K. D. Dudoit S., 2010 Evaluation of statistical methods for normalization and differential expression in mRNA-Seq experiments. BMC Bioinformatics 11: 94. 20167110 10.1186/1471-2105-11-94 PMC2838869

[bib5] Dillies M. A. Rau A. Aubert J. Hennequet-Antier C. Jeanmougin M., 2013 A comprehensive evaluation of normalization methods for Illumina high-throughput RNA sequencing data analysis. Brief. Bioinform. 14: 671–683. 22988256 10.1093/bib/bbs046

[bib6] Ding B. Zheng L. Zhu Y. Li N. Jia H., 2015 Normalization and noise reduction for single cell RNA-seq experiments. Bioinformatics 31: 2225–2227. 25717193 10.1093/bioinformatics/btv122 PMC4481848

[bib7] Dueck H. R. Ai R. Camarena A. Ding B. Dominguez R., 2016 Assessing characteristics of RNA amplification methods for single cell RNA sequencing BMC Genomics. 17: 966. 10.1186/s12864-016-3300-3 PMC5122016 27881084

[bib8] Dunn J. C., 1973 A fuzzy relative of the ISODATA process and its use in detecting compact well-separated clusters. J. Cybern. 3: 32–57.

[bib9] Dunn J. C., 1974 Some recent investigations of a new fuzzy partitioning algorithm and its application to pattern classification problems. J. Cybern. 4: 32–57.

[bib10] Garber M. Grabherr M. G. Guttman M. Trapnell C., 2011 Computational methods for transcriptome annotation and quantification using RNA-seq. Nat. Methods 8: 469–477. 21623353 10.1038/nmeth.1613

[bib11] Hennig C., 2007 Cluster-wise assessment of cluster stability. Comput. Stat. Data Anal. 52: 258–271.

[bib12] Jaccard P., 1912 a The distribution of the flora in the alpine zone. New Phytol. 11: 37–50.

[bib13] Kumar R. M. Cahan P. Shalek A. K. Satija R. DaleyKeyser A. J., 2014 Deconstructing transcriptional heterogeneity in pluripotent stem cells. Nature 516: 56–61. 25471879 10.1038/nature13920 PMC4256722

[bib14] Kurn N. Chen P. Heath J. D. Kopf-Sill A. Stephens K. M., 2005 Novel isothermal, linear nucleic acid amplification systems for highly multiplexed applications. Clin. Chem. 51: 1973–1981. 16123149 10.1373/clinchem.2005.053694

[bib15] Love M. I. Huber W. Anders S., 2014 Moderated estimation of fold change and dispersion for RNA-seq data with DESeq2. Genome Biol. 15: 550. 25516281 10.1186/s13059-014-0550-8 PMC4302049

[bib16] Morris J. Singh J. M. Eberwine J. H., 2011 Transcriptome analysis of single cells. J. Vis. Exp. 10.3791/2634 PMC3376915 21540826

[bib17] Mortazavi A. Williams B. A. McCue K. Schaeffer L. Wold B., 2008 Mapping and quantifying mammalian transcriptomes by RNA-Seq. Nat. Methods 5: 621–628. 18516045 10.1038/nmeth.1226 PMC13303166

[bib18] Patel A. P. Tirosh I. Trombetta J. J. Shalek A. K. Gillespie S. M., 2014 Single-cell RNA-seq highlights intratumoral heterogeneity in primary glioblastoma. Science 344: 1396–1401. 24925914 10.1126/science.1254257 PMC4123637

[bib19] Ramskold D. Luo S. Wang Y. C. Li R. Deng Q., 2012 Full-length mRNA-Seq from single-cell levels of RNA and individual circulating tumor cells. Nat. Biotechnol. 30: 777–782. 22820318 10.1038/nbt.2282 PMC3467340

[bib20] Rand W. M., 1971 Objective criteria for the evaluation of clustering methods. J. Am. Stat. Assoc. 66: 846–850.

[bib21] Risso D. Ngai J. Speed T. P. Dudoit S., 2014 Normalization of RNA-seq data using factor analysis of control genes or samples. Nat. Biotechnol. 32: 896–902. 25150836 10.1038/nbt.2931 PMC4404308

[bib22] Robinson M. D. Oshlack A., 2010 A scaling normalization method for differential expression analysis of RNA-seq data. Genome Biol. 11: R25. 20196867 10.1186/gb-2010-11-3-r25 PMC2864565

[bib23] Stegle O. Teichmann S. A. Marioni J. C., 2015 Computational and analytical challenges in single-cell transcriptomics. Nat. Rev. Genet. 16: 133–145. 25628217 10.1038/nrg3833

[bib24] Tan P.-N. Steinbach M. Kumar V., 2006 Introduction to Data Mining. Pearson Addison Wesley, Boston, MA.

[bib25] Trapnell C. Cacchiarelli D. Grimsby J. Pokharel P. Li S., 2014 The dynamics and regulators of cell fate decisions are revealed by pseudotemporal ordering of single cells. Nat. Biotechnol. 32: 381–386. 24658644 10.1038/nbt.2859 PMC4122333

[bib26] Treutlein B. Brownfield D. G. Wu A. R. Neff N. F. Mantalas G. L., 2014 Reconstructing lineage hierarchies of the distal lung epithelium using single-cell RNA-seq. Nature 509: 371–375. 24739965 10.1038/nature13173 PMC4145853

[bib27] Usoskin D. Furlan A. Islam S. Abdo H. Lonnerberg P., 2015 Unbiased classification of sensory neuron types by large-scale single-cell RNA sequencing. Nat. Neurosci. 18: 145–153. 25420068 10.1038/nn.3881

