# Supplementary material for: Assessment of Single Cell RNA-Seq Normalization Methods
Source: G3 (Bethesda). 2017 May 3;7(7):2039–45. doi: 10.1534/g3.117.040683 (PMC5499114; doi:10.1534/g3.117.040683)

**Supplementary Material**

**Figure S1. Hierarchical clustering of 120 samples with different normalization methods not using ERCC.** (a) DESeq; (b) UQ; (c) FPKM; (d)RUVr; (e) TMM. A, aRNA; N, Nugen Ovation RNASeq V2; S, C1 SMARTer.


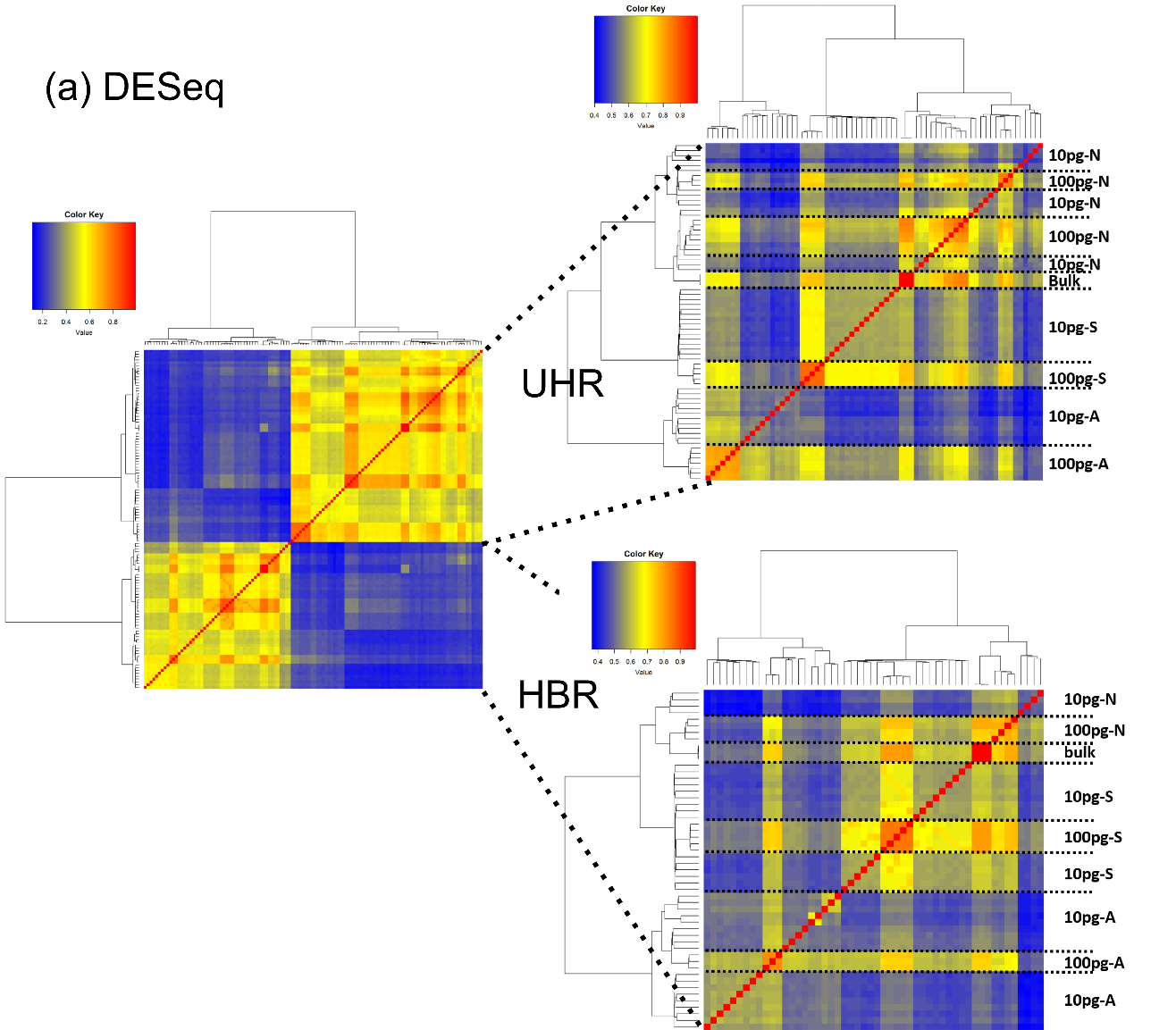


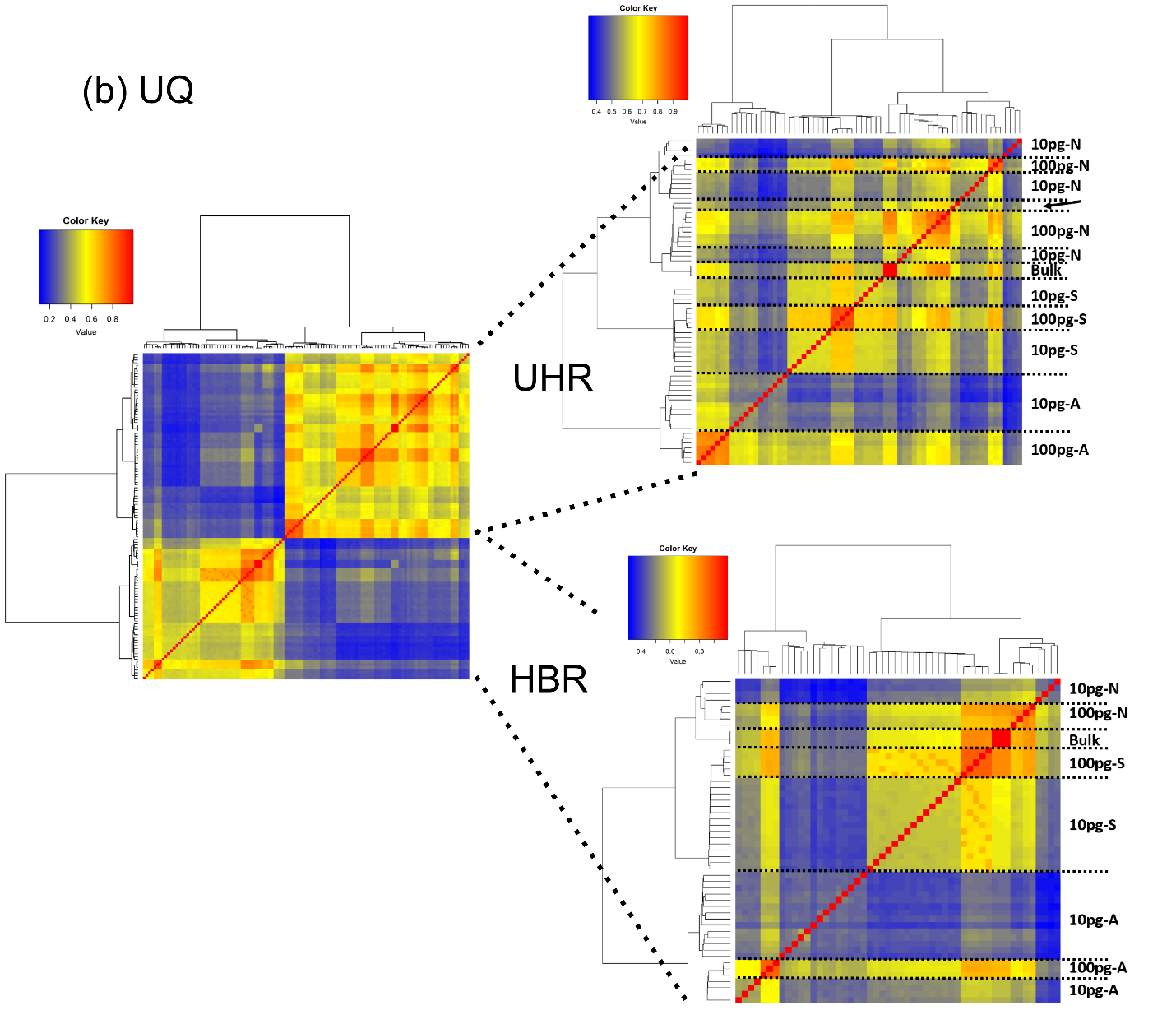


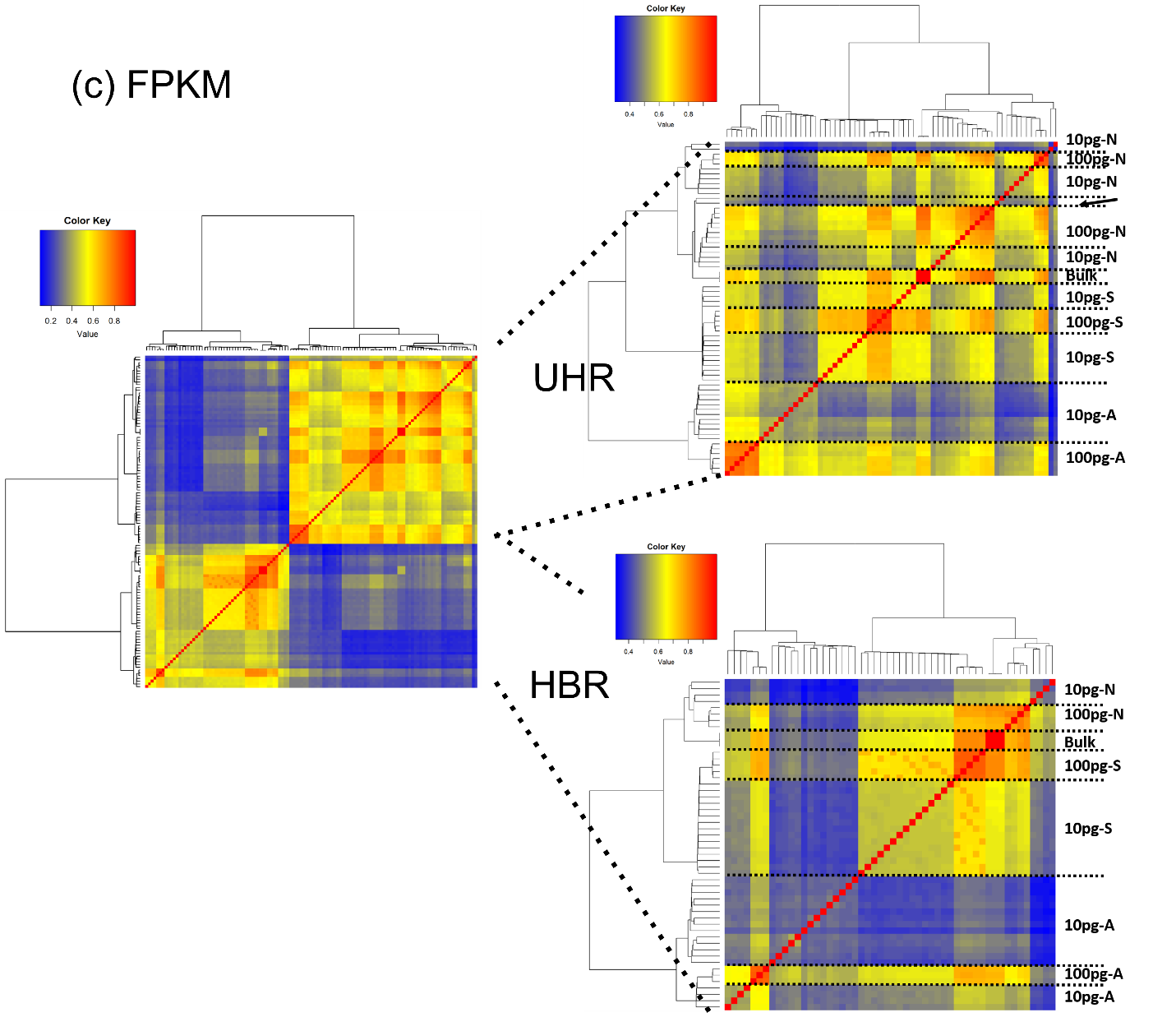


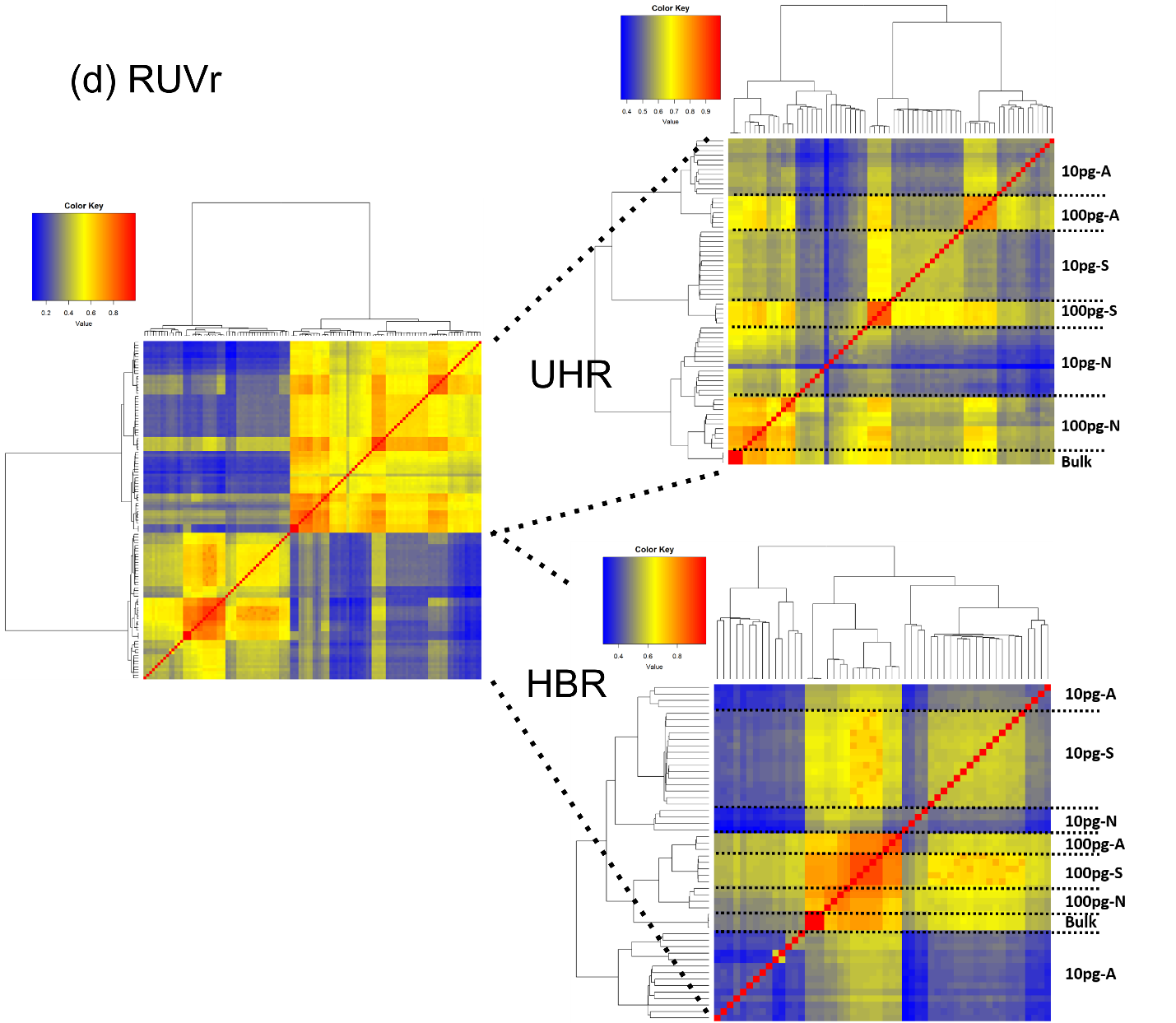


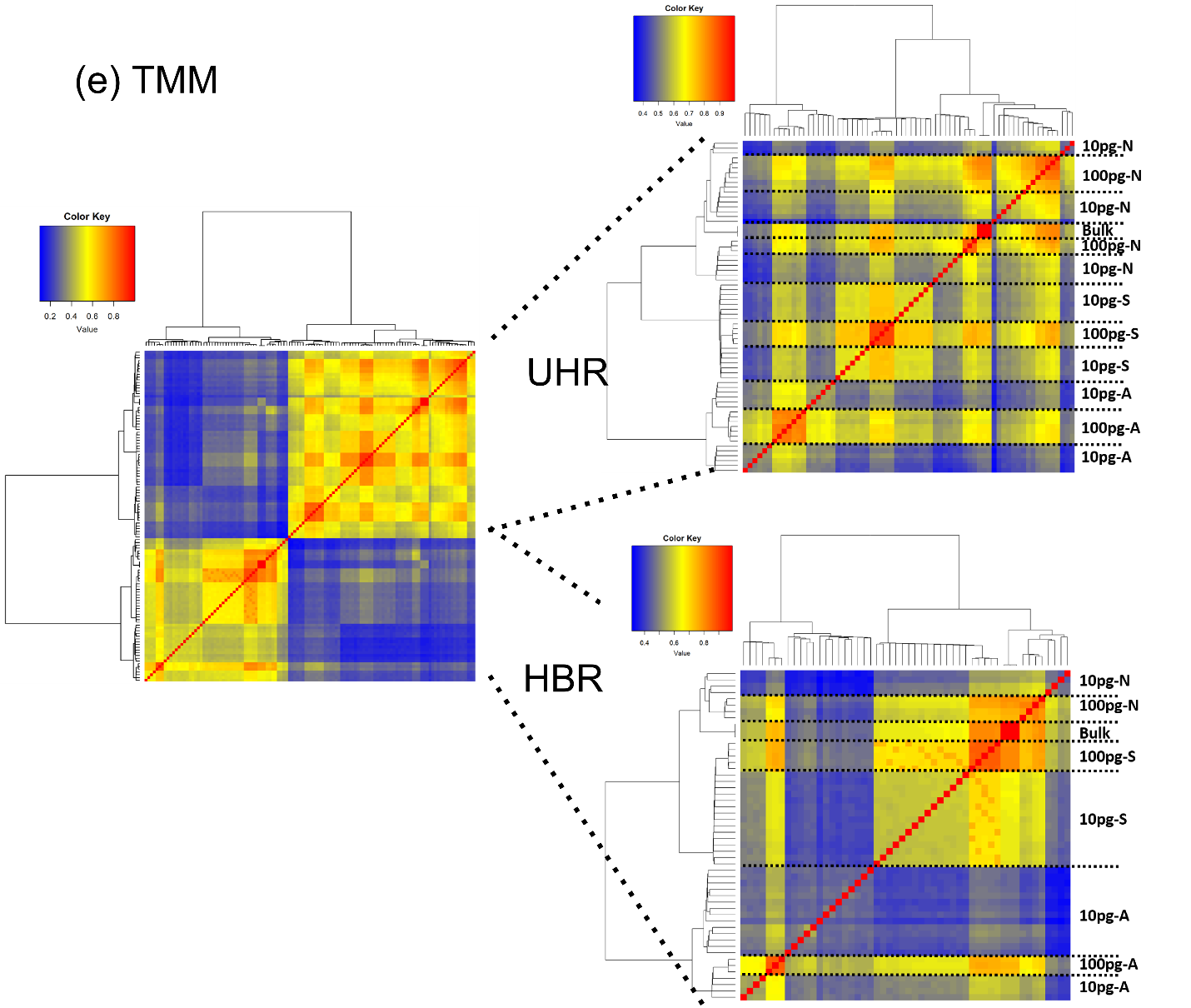


**Figure S2. Comparison of 5 normalization methods not using ERCC to cluster UHR samples.** (a) Rand index with different number of clusters; (b) Dunn index with different number of clusters; (c) Jaccard index with three clusters; (d) Rand index with the most variable genes; (e) Rand index with the least variable genes (f) Dunn index with the most variable genes; (g) Dunn index with the least variable genes. (d)-(g) are results using 3 clusters as the ground truth (bulk, 100pg and 10pg UHR samples).
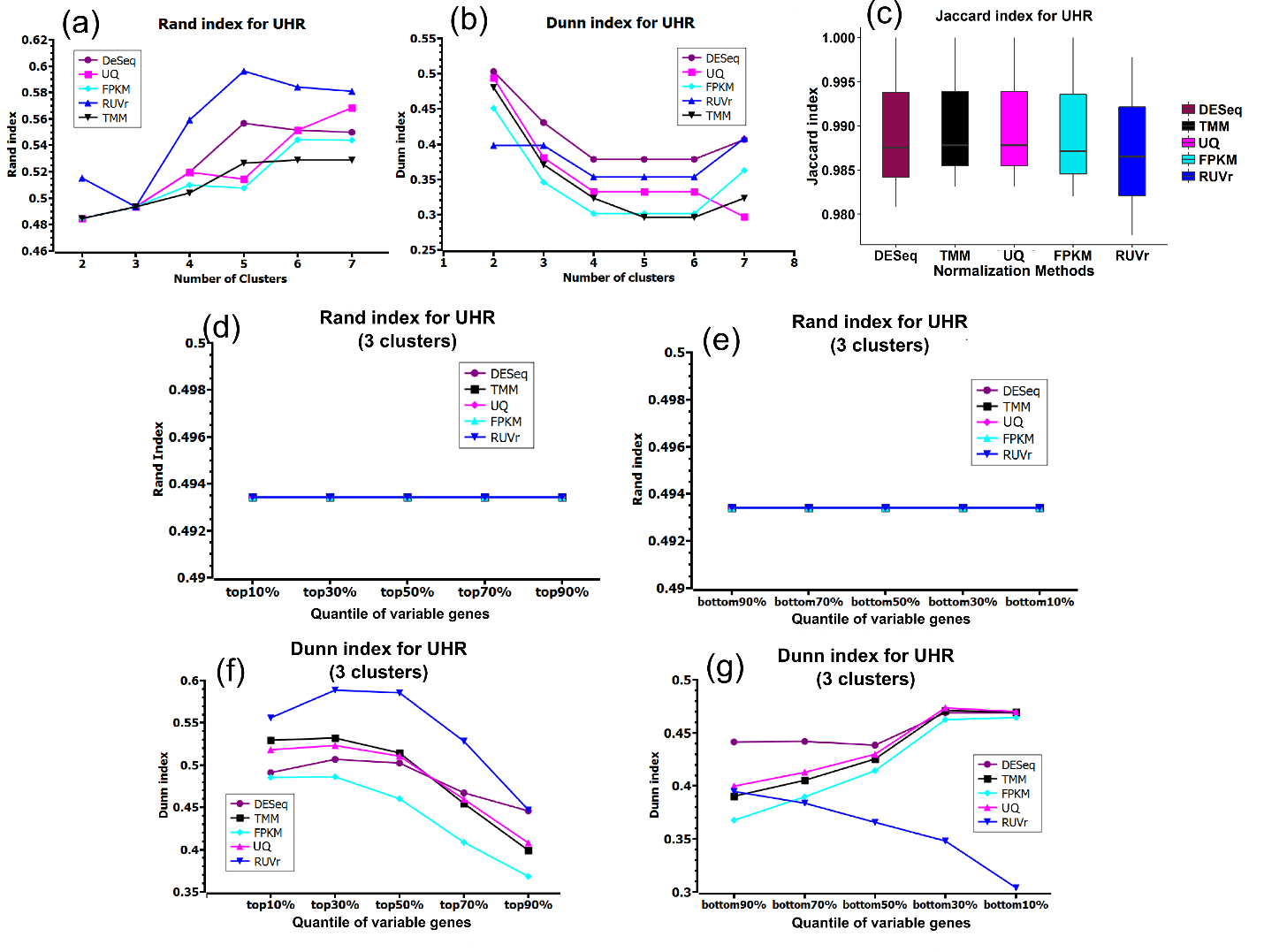


**Figure S3. Jaccard index for samples normalized by GRM, RUVg and RUVr and clustered to 6 clusters.** The ground truth is 6 clusters: HBR with 10pg, 100pg and bulk; UHR with 10pg, 100pg and bulk. Jaccard index for samples normalized by GRM, RUVg and RUVr using different sets of genes ranked by their variability across samples. (a) Different quantiles of top variable genes; (b) Different quantiles of least variable genes.


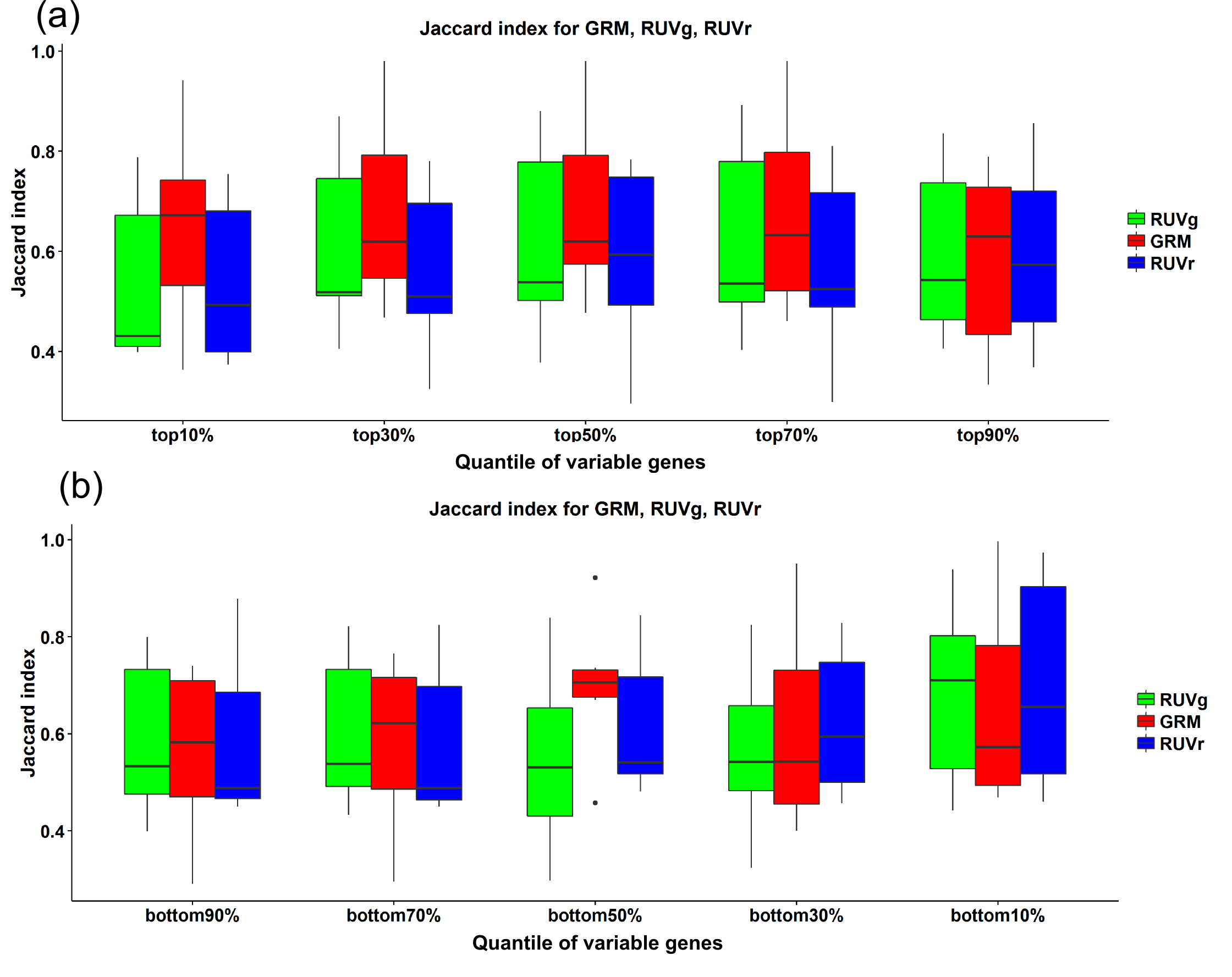

Supplement: Supplementary file 1 [file 2039FileS1.docx]
